# Exposure to non-endemic arboviruses (alphaviruses) in Costa Rica assessed from human samples collected in areas with contrasting levels of dengue endemicity

**DOI:** 10.3389/fpubh.2025.1537019

**Published:** 2025-02-19

**Authors:** Andrea Valles-Morera, Tatiana Murillo, Jose Lizano-Bolaños, Sergio Gutierrez-Roche, Margarita Alvarado, Jonathan Alfaro-Alvarado, Gerardo Andrés Calvo-Salas, Grace Prado-Hidalgo, Johis Ortega, Eugenia Corrales-Aguilar

**Affiliations:** ^1^Virology-Research Center for Tropical Diseases (CIET), Faculty of Microbiology, University of Costa Rica, San José, Costa Rica; ^2^Department of Biology, University of Miami, Coral Gables, FL, United States; ^3^Blood Bank and Clinic Laboratory, University of Costa Rica, San José, Costa Rica; ^4^Talamanca Healthcare Center, Costa Rican Social Security Fund, Limón, Costa Rica; ^5^School of Nursing and Health Studies, University of Miami, Coral Gables, FL, United States

**Keywords:** arboviruses, alphaviruses, serology, Venezuelan equine encephalitis virus, neutralization

## Abstract

Arboviruses represent a global public health challenge. The lack of diagnostic protocols and the presence of asymptomatic infections complicate confirmatory diagnostics. Alphaviruses, such as the equine encephalitis viruses, can cause severe outbreaks and are usually misdiagnosed as dengue. Thus, evidence for their circulation was assessed here. Plaque reduction neutralization test (PRNT) was used to compare sera collected during 2022–2023 from an area with high dengue endemicity (Hone Creek) with another with low endemicity (Great Metropolitan Area, GMA) to elucidate the putative alphavirus circulation and determine whether there were differences between the two areas. The screening results of PRNT50% against the Venezuelan equine encephalitis virus (VEEV) and the Eastern equine encephalitis virus showed that 20.5% of sera collected from Hone Creek were positive for VEEV, with 15.4% (*n* = 40) showing real neutralizing titers. In the GMA, only 0.8% tested positive for VEEV during the screening, with only 0.3% (*n* = 1) showing a true neutralizing titer. No sample was positive for the Eastern equine encephalitis virus or Mayaro (MAYV) and one serum sample from Hone Creek was chikungunya positive. This study underscores the global health challenge posed by arboviruses with their similar clinical presentation and antibody cross-reactivity, particularly in tropical regions where flaviviruses and alphaviruses prevail and co-circulate. The comparison of PRNT results between high and low dengue-endemic areas in Costa Rica shed light on the potential circulation of the VEEV and the fact that there is no circulation of Eastern equine encephalitis virus or Mayaro yet. These findings indicate a higher prevalence of VEEV in the high-endemic area, emphasizing the importance of targeted surveillance, control measures, and better diagnostics.

## 1 Introduction

Arboviruses, arthropod-borne viruses, are zoonotic viruses transmitted by arthropods. They include the *Bunyaviridae*, *Flaviviridae,* and/or *Togaviridae* families. The *Togaviridae* comprises the genus *Alphavirus* with clinically important viruses such as Venezuelan equine encephalitis virus (VEEV), chikungunya (CHIKV), Mayaro, and Eastern equine encephalitis virus (EEEV) (1, 2). The primary vectors for these alphaviruses are mosquitoes from the genera *Aedes*, *Culex*, *Culiseta*, and *Haemagogus,* and the main reservoirs are small mammals such as *Sigmodontinae*, *Oryzomys*, and *Didelphis marsupialis* (3, 4), except for CHIKV, where human–mosquito–human urban and/or peri-urban transmission takes place without a known urban reservoir involved (5, 6). The viral cycle of VEEV and EEEV consists of transmission between these reservoirs and mosquitoes, with accidental exposure to humans (and other mammals). The clinical presentation of many alphaviruses ranges from asymptomatic to severe diseases (7, 8). Of interest, many infections are accounted to be asymptomatic, with CHIKV having the lowest percentage of asymptomatic cases (28%) (5, 9–12), in comparison with EEEV with 96% of asymptomatic cases (13). When symptomatic, both viruses can cause a febrile-like disease, although CHIKV causes mainly arthralgia, on the other hand, the equine encephalitis viruses are known to have high mortality (50–90%) rates or severe neurological sequelae in patients with any neurological symptoms (7, 8, 14). Thus, in the public health context, detecting exposure through serological studies is essential to determining the possible local circulation of these non-endemic arboviruses.

On the American continent, several countries have detected the circulation of arboviruses transmitted by mosquitoes (4, 7, 8, 15–20). Of note, DENV and Oropouche in Central and South America have been reported to have caused thousands of infections during this year (2024) (21–23). Alphaviruses are not equally distributed throughout the whole continent. The EEEV can be found in a large part of North America, Central America, South America, and the Caribbean islands (1–3); the VEEV is reported in Central America and some regions of North America, South America, and Trinidad (4, 7–9); and the Western equine encephalitis virus (WEEV) has been mainly described in North America and South America (24, 25); nonetheless, infections have been on the rise in South America since November 2023 with human cases reported in Argentina (25). Most outbreaks of equine encephalitis reported in America are linked to horses. However, there is evidence of infection and disease in humans. For example, Vittor et al. (19) reported the presence of human neutralizing antibodies against VEEV and MADV (Madariaga virus)/EEEV in 31.5 and 4.8%, respectively, of inhabitants from the Darién area, Panama. Similarly, in 2017, Carrera et al. (20) studied samples from the same area and detected the presence of neutralizing antibodies against MADV/EEEV (13.2%), VEEV (16.8%), and Mayaro virus (MAYV) (1.2%). Costa Rica is considered endemic to humans for some arboviruses (26), with DENV presenting the highest number of annual cases (16). Other arboviruses, such as VEEV and MADV/EEEV, have been reported as endemic in horses (27–29) and detected in other animals, such as bats and birds (3, 4, 29, 30). A previous study conducted in the 1970s showed a human prevalence of 10% (n = 155) for VEEV antibodies in Guanacaste (29). However, they are not considered human endemic viruses here or in neighboring countries (19) and 11 human cases, 3 severe, and 1 mortal have been identified in different regions of Costa Rica (16–18, 28, 31, 32).

Diagnostics for viral encephalitis and meningoencephalitis are complex. To confirm a case of viral presence via molecular methods or viral isolation, specific IgM in CSF, or seroconversion, paired samples in serum or CSF must be measured. These viruses produce low viremia and a short replication time in humans (except for CHIKV), and the viremia peak usually occurs before the appearance of symptoms, thus making molecular diagnostics challenging and frequently producing false-negative results (20, 33–35). Therefore, serological techniques, such as enzyme-linked immunosorbent assay (ELISA) and the detection of neutralizing antibodies by plaque reduction viral neutralization technique (PRNT), are essential for diagnosis and epidemiological monitoring (34, 35). Serological discrimination between two (or more) different alphaviruses is a problem as cross-reactivity is high and this can also lead to incorrect diagnosis (34, 35). Thus, PRNT is considered the gold standard for *Alphavirus* genre diagnosis, as it not only shows high specificity but also allows the *in vitro* determination of the neutralizing capacity of the antibodies detected (10, 35). Cross-reactivity represents a greater challenge in countries with high endemicity for alphaviruses or in regions where there is also co-circulation of two or more arboviruses. It is hypothesized that many of the VEEV outbreaks in America have been misdiagnosed as dengue or influenza (4, 30) due to their similar clinical presentation and the incorrect use of diagnostic tools (4, 7, 30, 34).

In Costa Rica, laboratory-based arbovirus surveillance is carried out by the INCIENSA (Costa Rican Institute of Research and Teaching in Nutrition and Health) detecting DENV, CHIVK, and ZIKV. Then, molecular biology is used in negative samples to detect WNV, MAYV, and YFV, but not against VEEV nor MADV/EEEV (16, 36). Hence, currently, there is no broad monitoring of human infections caused by alphaviruses in the country. Therefore, we aimed to investigate the circulation of non-endemic alphaviruses to determine their possible co-circulation with other arboviruses in Costa Rica by testing the seropositivity against alphavirus in human samples from high endemic vs. low endemic reported areas of arbovirus circulation.

## 2 Materials and methods

### 2.1 Human samples

Alphavirus exposure was evaluated in human serum samples obtained from the Hone Creek Integral Attention Health Centre in Talamanca (n = 259) and the Trauma Hospital Blood Bank in the Greater Metropolitan Area (GMA) (n = 367). These two areas were selected for high or low dengue transmission reported by the National Surveillance System (16, 21). Samples were randomly selected after obtaining consent from volunteers to donate blood. These sera were anonymized and transported at 4°C to the Virology Laboratory at the University of Costa Rica’s Microbiology Faculty. The University of Costa Rica Ethic Scientific Committee (CEC/IRB) approved using human sera samples according to the principles expressed in the Declaration of Helsinki (VI-3178-2017, VI-5898-2017, VI-1542-2018). In addition, it was part of the community outreach project ED-3257.

### 2.2 Virus strains

The alphavirus strains used were the SINV/MADV (BeAn436087, also known as the South American strain, isolated in Brazil for MADV/EEEV) and the VEEV (TC 0083, 252,296), both donated by the Gorgas Memorial Institute of Health; the 181/25 CHIKV vaccine strain and MAYV/IRES vaccine strain, both donated by the World Reference Center for Emerging Viruses and Arboviruses from the University of Texas Medical Branch (19, 37–39). Furthermore, yellow fever (YFV) chimeric viruses expressing DENV-1 envelope protein (YFV 17D/DENV-1 PUO 359) and/or the Saint Louis encephalitis virus (SLEV) envelope protein (YFV 17D/SLEV CorAn 9,124) donated by the Center for Disease Control and Prevention were used.

Briefly, viruses were grown in VERO cells (ATCC: CCL-81) with minimum essential medium (MEM Sigma® M0769) supplemented with 5% fetal bovine serum (FBS Sigma® F4135) for 72 h at 37°C in a 5% CO_2_ atmosphere. After the cytopathic effect (CPE) was evident and the incubation time ended, supernatants were collected, centrifuged, aliquoted, and stored at −80°C. The strains were titrated in 48-well plates containing monolayered VERO cells to obtain plaque-forming units per mL (PFU/mL).

### 2.3 Serological screening for flavivirus antibodies with PRNT

For dengue seroprevalence, PRNT was done as previously described (37). Percentages for each area were calculated to determine high and low dengue prevalence. PRNT was conducted using yellow fever (YFV) chimeric viruses expressing DENV-1 envelope protein or (SLEV) envelope protein. Briefly, sera were heat-inactivated at 56°C for 30 min and used for screening at a 1:20 dilution minimum essential medium (MEM) with 2% fetal bovine serum and mixed with an equal volume of each virus. The virus–antibodies mix was incubated for 1 h at 37°C in a 5% CO_2_ atmosphere, and a 100 μL volume was inoculated into a Vero (ATCC CCL-81) cell monolayer afterward. Then, it was removed, and MEM with 1% carboxymethylcellulose was added. After 5 days of incubation, plates were fixed with formalin (3.7%) for an hour and stained with crystal violet (1%). Sera that resulted in 90% neutralization relative to the average of the viral control (no sera) were considered DENV-1 or SLEV positive.

### 2.4 Serological screening of VEEV antibodies by PRNT

Sera samples collected were heat-inactivated at 56°C for 30 min, then diluted 1:20 in MEM with 2% of FBS and mixed with the respective virus strain to an estimated 15 PFU/well result. The mix was incubated at 37°C in a 5% CO_2_ atmosphere for an hour and then placed in VERO cells. Following a 1-h incubation, the inoculum was removed, and each well was supplemented with 1.5% carboxymethylcellulose (CMC, Sigma® C4888) and 2% FBS. Plates were incubated for 96 h at 37°C in a 5% CO_2_ atmosphere. Afterward, cells were fixed using 3.7% formalin and stained using 1% crystal violet. Sera that resulted in 50% neutralization relative to the average of viral control (no sera) were considered alphavirus positive.

### 2.5 Sera titration against VEEV, MADV/EEEV, CHIKV, and Mayaro with PRNT

Sera that resulted in reactive against VEEV at 1:20 dilution in the screening were then titrated against VEEV, MADV/EEEV, CHIKV, and Mayaro. To do so, each sample was processed in triplicate, and each was diluted following serial 2-fold dilutions ranging from 1:20 to 1:1280, or higher if needed, according to the same protocol as performed during the screening. A plaque reduction of ≥50% compared to the average of the viral control was considered positive against the virus strain evaluated. Furthermore, the highest dilution showing a reduction of ≥50% of the virus was considered the serum titer for each tested sample. The cutoff of PRNT50% was used as neither VEEV, MADV/EEEV, CHIKV, nor Mayaro are considered endemic human viruses in Costa Rica (19, 37). Finally, a ≥ 4-fold difference in the titers among viruses was considered sufficient to classify the sample studied as unequivocally positive, showing the presence of neutralizing antibodies against the virus with the highest titer (37). In contrast, a difference of less than 4-fold dilutions was considered non-specific and classified as positive against Alphavirus, though indeterminate, as it is probably the result of cross-reaction against another Alphavirus or other arboviruses not evaluated in this study (37).

### 2.6 Statistical analysis

All statistical analyses were performed in R with R Studio (40, 41), and the results were visualized with ggplot2 (42). A chi-square test (after confirming the data did not include frequencies lower than 5) was used to determine whether there were significant differences in the number of positive samples for neutralizing antibodies against alphaviruses and, of note, VEEV.

## 3 Results

### 3.1 Serological screening by PRNT shows a higher number of positive arbovirus samples in the high dengue-endemic area than in the low-endemic area

**Figure 1:**
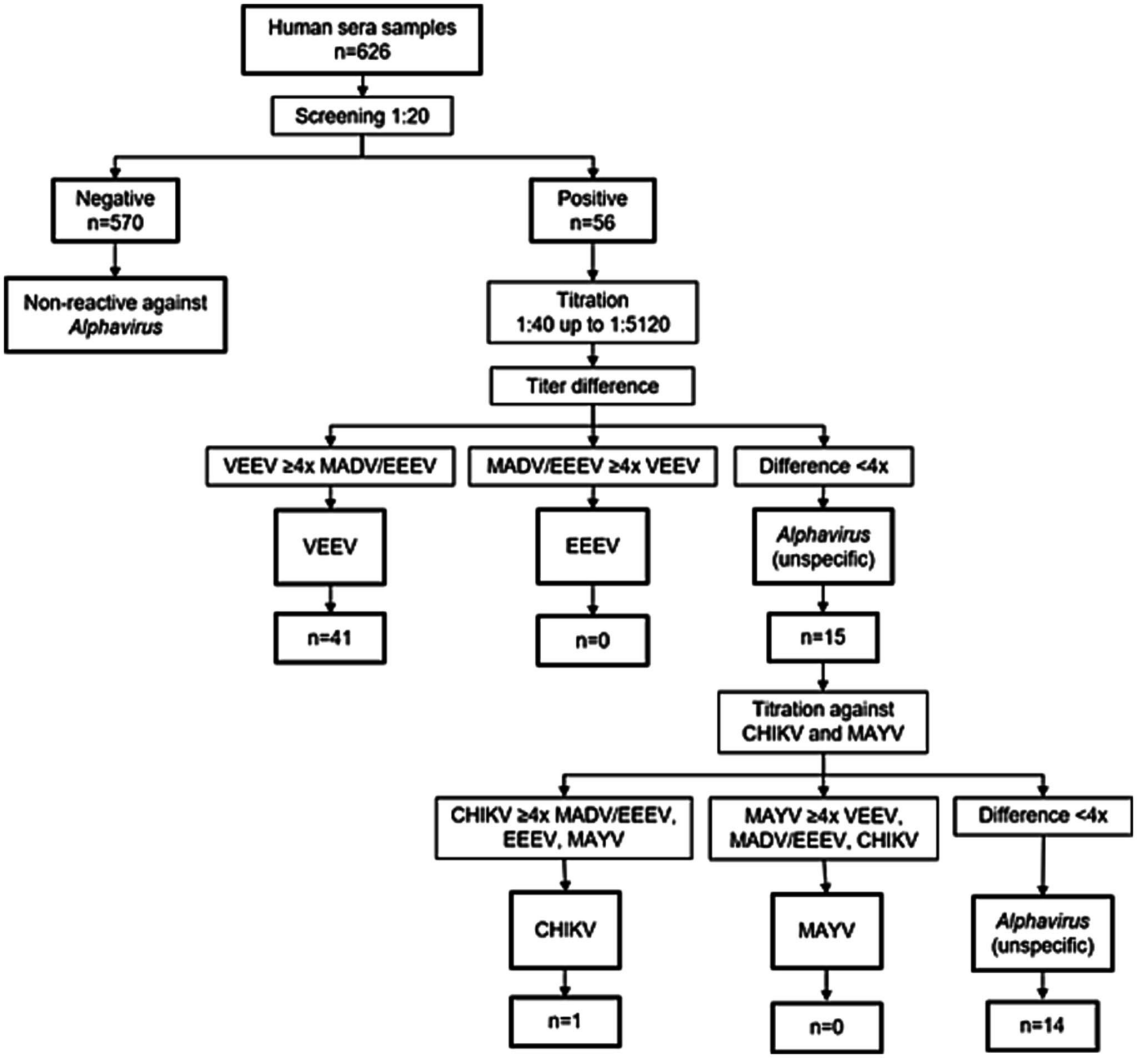
Algorithm used for the sera classification regarding neutralizing antibodies against alphaviruses from both Talamanca and GMA human sera samples. The value 4x refers to a 4-fold difference between the two viruses analyzed, and a difference equal or greater between the titers was used to classify a given serum as unequivocally positive against a specific virus. When there was a difference of less than 4-fold between the titers, the term ‘unspecific’ was used for a given serum. VEEV: Venezuelan equine encephalitis virus; MADV/EEEV: Eastern equine encephalitis virus; CHIKV: chikungunya virus, MAYV: Mayaro virus.

PRNT was done to verify dengue endemicity. Hone Creek sera depicted more than 80% for DENV-1 and SLEV positivity, contrasting with GMA sera with less than 15%, confirming the high and low areas of flavivirus prevalence. The comparative serological screening for the alphaviruses study was carried out using human sera from Talamanca (Hone Creek) and GMA (Greater Metropolitan Area) by PRNT50% against VEEV, as shown in Figure 1.

As depicted in Table 1, 259 sera from Talamanca were screened against VEEV, 53 (20.5%) samples were identified as positive (reactive), and 206 (79.5%) samples were classified as non-reactive at a serum dilution of 1:20. On the contrary, out of 367 sera from the GMA analyzed, only 3 (0.8%) samples were identified as alphavirus-positive (reactive) and 364 (99.2%) as alphavirus-non-reactive. As expected, screening results show a higher percentage of positive seroprevalence against alphaviruses in the region of Talamanca (high DENV endemicity) than GMA (low DENV endemicity).

**Table 1:** Human sera samples screened against VEEV using plaque reduction neutralization test (PRNT 50%).

	Talamanca		GMA	
	*N*	%	*n*	%
Number of samples screened	259	100.0	367	100.0
Non-reactive samples	206	79.5	364	99.2
Reactive samples	53	20.5	3	0.8

VEEV, Venezuelan equine encephalitis virus.

### 3.2 Titration of alphavirus-reactive samples to determine specificity shows the circulation of VEEV in a high dengue endemic area

Samples positive in screening against VEEV were titrated in serial 2-fold dilutions against VEEV (Figure 1). Of the three human sera samples from the GMA titrated, only one demonstrated an unequivocally positive titer against VEEV. At the same time, the other two were classified as alphavirus unspecific or indeterminate. Alternatively, out of 53 titrated samples from Talamanca, 40 (15.4%) showed an unequivocal neutralizing titer against VEEV, and 13 (5.0%) samples were classified as unspecific-positive for Alphavirus (Table 2). The alphavirus-unspecific labeled samples from both Talamanca and GMA were also tested against MADV/EEEV, CHIKV, and MAYV. After antibody titration against these other alphaviruses, only one of these samples presented specific CHIKV-neutralizing antibodies with a titer of >1:1280. However, none of the human sera samples were unequivocally positive against MADV/EEEV. In addition, no positives against Mayaro were detected. Therefore, we cannot confirm or discard the current circulation of MADV/EEEV or Mayaro so far in Costa Rica in this study.

**Table 2:** Sera samples titrated against VEEV and MADV/EEEV using the plaque reduction neutralization test (PRNT 50%).

	Talamanca		GMA	
	*n*	%	*n*	%
Human sera samples reactive in the screening	53	20.5	3	0.8
VEEV-positive samples	40	15.5	1	0.3
MADV-/EEEV-positive samples	0	0.0	0	0.0
*Alphavirus-*positive samples (unspecific)	13	5.0	2	0.5

VEEV, Venezuelan equine encephalitis virus; MADV/EEEV, Eastern equine encephalitis virus.

All samples were serially titrated by PRNT 50% starting at 1:20 and thoroughly diluted to determine each sample’s antibody titer against VEEV. Out of the total of unequivocally positive samples against VEEV, 40 human sera samples from Talamanca presented titers between 1:80 and 1:5120 (Supplementary Table S1.1); meanwhile, the only unequivocally positive sample from GMA showed a titer of 1:160 (Supplementary Table S1.2). These results confirm the high presence of neutralizing antibodies against VEEV in Talamanca. It is tempting to speculate that the high antibody titer indicates more virus circulation or recent exposure to the virus.

### 3.3 Statistical analysis shows significant differences in alphavirus seroprevalence between a high and a low dengue-endemic area

**Figure 2:**
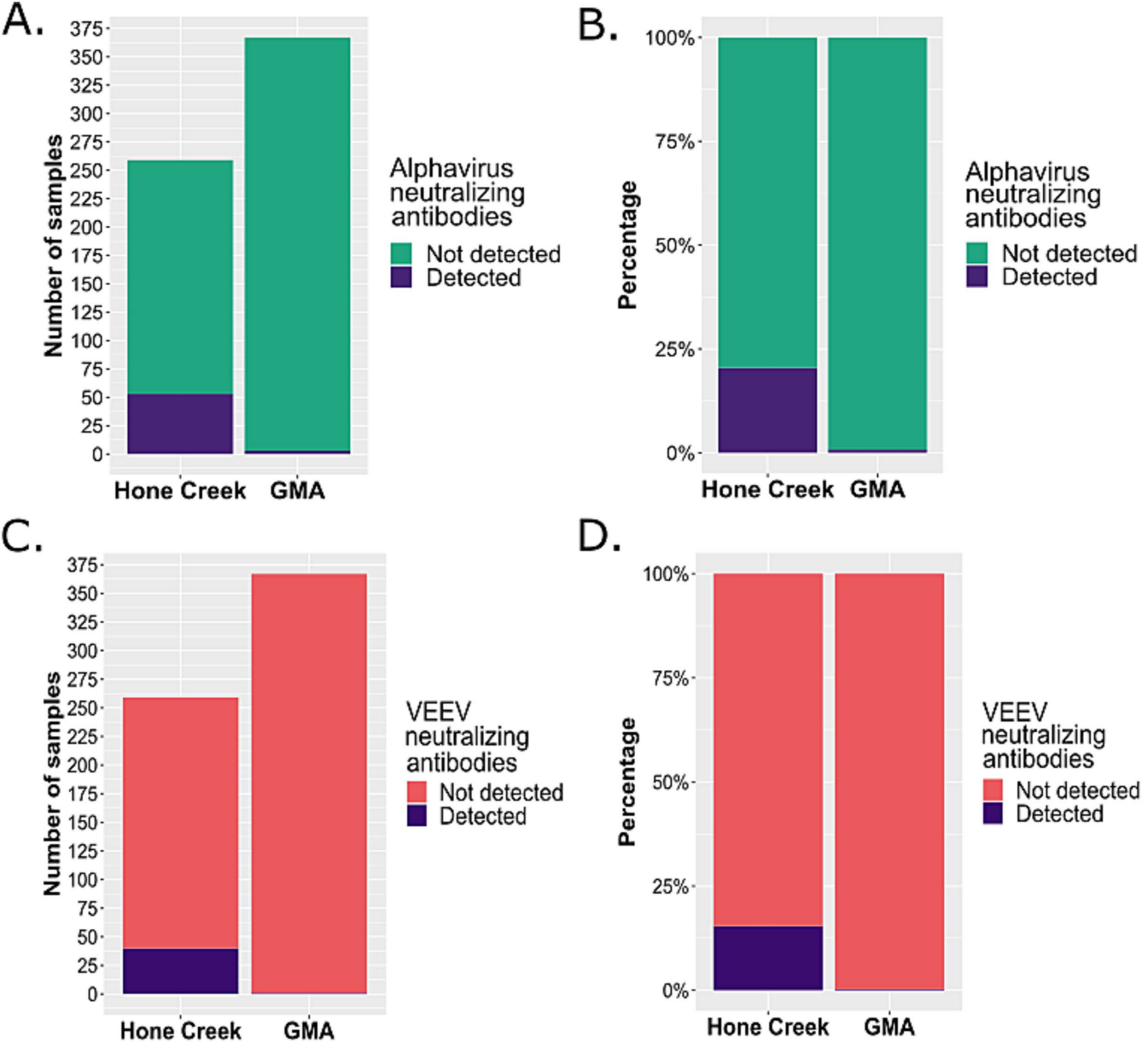
Total number and percentage of samples from endemic and non-endemic regions for arboviral circulation in Costa Rica unequivocally positive against **(A,B)** alphaviruses and **(C,D)** VEEV. Serum samples were collected in an endemic region (Hone Creek) and non-endemic region (GMA) and were tested with PRNT 50% for the detection of antibody titers against VEEV. A chi-squared test confirmed there are significant differences between the number of detected positives and the sampling site for alphavirus-neutralizing antibodies (*χ*^2^ = 69.6 and *p*-value <0.05) and VEEV-neutralizing antibodies (*χ*^2^ = 54.7 and *p*-value <0.05).

The chi-square test was performed for statistical comparisons of the detection of neutralizing antibodies against Alphavirus and VEEV in endemic and non-endemic arbovirus circulation regions in Costa Rica. The test confirmed significant differences between the number of detected positives against alphaviruses at each sampling site (*χ*^2^ = 69.6 and *p*-value <0.05). The residuals show that the correlation of positive samples was negative for GMA and positive for Hone Creek (Supplementary Table S2). In addition, significant differences between the number of detected positives against VEEV and each sampling site were detected (*χ*^2^ = 54.7 and p-value <0.05, Figure 2). The residuals showed that the correlation of positive samples was negative for GMA and positive for Hone Creek (Supplementary Table S2). Interestingly, some of the samples were cross-reactive. We detected a specific anti-CHIKV positive person in Talamanca with a titer of >1:1280 (Supplementary Table S1.1), but no specific Mayaro antibodies were found so far. Summing up, these results show that viruses of the *Alphaviridae* family, such as VEEV, do circulate in humans in our country, especially in areas with a high number of cases of other arboviral infections, such as DENV. These findings imply virus transmission and the presence of putative reservoirs and vectors in a high diversity and mosquito density area, as described by Romero Vega et al. (43).

## 4 Discussion

Even though alphaviruses in Costa Rica are linked to a high number of infections in animals, only CHIKV is considered here so far circulating in humans. This research showed evidence of neutralizing antibodies in human sera samples against alphaviruses, with a higher seroprevalence in the Hone Creek region than the GMA. This hints at a possible circulation of these viruses in our country and, interestingly, with a higher distribution in a region near Panamá, where reports of these alphaviruses exist (19, 20, 44). Our results coincide with previous reports of VEEV and MADV/EEEV circulation in animals, such as horses, bats, and birds in Costa Rica (3, 29). León et al. (27) reported the presence of equine neutralizing antibodies against VEEV and MADV/EEEV along all the provinces of Costa Rica (n = 217), with the highest seropositivity in Limón (PRNT80%), the same province/region where Hone Creek is located. Furthermore, Costa Rica is endemic to *Aedes albopictus* and *Aedes aegypti*, which are vectors of DENV, ZIKV, CHIKV, MAYV, VEEV, and EEEV, as well as *Culex* (*Melanoconion*) and *Deinocerites pseudes* (37, 43), both confirmed vectors for VEEV (45, 46). The presence of some alphavirus reservoirs, putative vectors, and our specific serological findings confirm that alphaviruses are circulating in our country, which might lead to an increased risk of human infection, progression to disease, and death.

The presence of human neutralizing antibodies against alphaviruses in Costa Rica is not surprising, as there are reported present and past circulation of VEEV, MADV/EEEV, MAYV, and CHIKV in neighboring countries such as Panamá and Nicaragua (4, 15–17, 30). Vittor et al. (19) reported a seroprevalence of 4.81% (n = 770, PRNT80%) against MADV and a seroprevalence of 31.34% (n = 769, PNT80%) against VEEV in the Darién region, Panama. Carrera et al. (20) demonstrated seropositivity in the same region of Darién against MADV/EEEV (13.2%), VEEV (16.8%), and MAYV (1.2%) using PRNT80% (n = 243), along with one human death confirmed to be caused by MADV/EEEV. A more recent exposure later than in the 70s (29) or higher circulation is suggested from the titers obtained in Hone Creek (1:80–1:5120). There are reports of the association of VEEV seropositivity in humans and working in rural sites with proximity to forest coverage, both conditions present in Hone Creek (8, 19, 27, 28). Therefore, the evidence of a higher number of positive samples against alphaviruses in Hone Creek than GMA could be explained by the presence of reservoirs and/or vectors and cross-country dissemination of the viruses between Costa Rica and Panamá.

The presence of neutralizing antibodies against MADV/EEEV was not detected in any samples, suggesting no evidence of MADV/EEEV circulation so far in the investigated areas. However, as our sampling was limited, we must keep the current circulation and active infections in humans and other animals elsewhere in Costa Rica. There have been reports of the circulation of MADV/EEEV in animals (3), human symptomatic infections in the country (16–18, 31), and evidence of human infections in Darien (Panamá) close to Hone Creek (19, 20). Therefore, we think MADV/EEEV seroprevalence should be investigated further when studying alphaviruses, as it may become a future challenge for local healthcare facilities.

Neutralizing antibodies against alphaviruses other than VEEV and MADV/EEEV were also detected. Further testing confirmed that one of the samples was positive for antibodies against CHIKV. However, 14 samples were classified as “unspecific Alphavirus,” meaning it was impossible to confirm against which virus the neutralizing antibodies were directed. Many infections caused by alphaviruses tend to be asymptomatic, and the low percentage with a clinical usually produces a febrile illness with flu-like symptoms, which is shared among arboviruses (5, 9, 10). Therefore, it is not uncommon for these infections to be misdiagnosed. Carrera et al. (20) also demonstrated the presence of neutralizing antibodies against MAYV and UNAV (Una virus, another alphavirus) in Darien (Panamá), showing the existence of seropositivity against other alphaviruses different from VEEV and CHIKV. Thus, other viruses that were not tested may also be circulating.

Cross-reactivity between arboviruses is of great importance in endemic areas such as Central America due to the evident co-circulation of flaviviruses (DENV and ZIKV) and alphaviruses (CHIKV) in humans and animals (16, 47). In the research by Barrantes Murillo et al. (3), a 39.3% cross-reaction between alphaviruses (VEEV and MADV/EEEV) and flaviviruses (DENV-1, DENV-2, DENV-3, DENV-4, and WNV) was reported when evaluating the neutralizing titer in bats (n = 144) from Costa Rica (PRNT 90%). In comparison, only 17.88% presented unequivocally positive titers against any of the before-mentioned viruses. Due to this evident co-circulation, IgM and IgG ELISA tests are not recommended to detect arboviruses, as cross-reactivity reactions, even against other pathogens, are common, mainly when testing with IgM (10, 34, 47). The confirmation of acute infections should be done using molecular techniques, with the constraint of having many false-negative results due to the low viremia caused by alphaviruses (7, 11, 17). This can also account for the low number of alphavirus infections reported in this area and the country (26). The gold standard for serological testing is PRNT, as it allows distinguishing between neutralizing antibodies against specific viruses; thus, it should be used for serological confirmation of cases of alphaviruses in Costa Rica and other arbovirus-endemic regions (10, 34, 35). Nonetheless, the PRNT technique can also have some disadvantages: expert laboratory skills are needed, there is a long waiting time (4–6 days) for results, it is expensive, the number of samples that can simultaneously be analyzed is usually low in comparison with ELISAs, and for many of these viruses, type 3 biosafety laboratory facilities are needed when not working with chimeric viruses (8, 20, 47, 48). These can make diagnosing and monitoring alphaviruses in countries with limited resources and facilities extremely difficult.

This serological study has some limitations: first, seropositivity was evaluated against only some viruses of the *Togaviridae* family. Therefore, the circulation of other alphaviruses different from VEEV, MADV/EEEV, CHIKV, and MAYV could be suspected, and the evaluation of the sera against other alphaviruses present in America, such as WEEV, is recommended (24). Second, it only determines the presence of antibodies in two areas of Costa Rica, which does not imply a national circulation or the absence of a country-wide circulation of these viruses. Additionally, it was limited to using sera from adults over 18 years of age. It would be interesting to determine the approximate age of seroconversion through age ranges to determine at what age exposure occurs. In addition, this study should be repeated as soon as possible, increasing the number of sera analyzed in order to monitor the evolution of the circulation of these viruses.

Furthermore, extensive studies on the presence of vectors and reservoirs and the positivity of these viruses in these areas would be valuable. Future vaccinations need to know baseline serology for many of these viruses, for example, DENV, even in high endemic areas, to make an educated decision about vaccination (when available). Nevertheless, detecting this high number of seropositive individuals and these high titers against some alphaviruses should inform the authorities of transmission events to establish guidelines for control, diagnosis, and prevention of deaths.

Costa Rica presents a high endemicity for DENV and other arboviruses. When infections with these viruses are diagnosed using clinical features or low-specificity tests, such as ELISA for IgM detection, this could mask the circulation of other arboviruses (8, 29). This research demonstrated the presence of neutralizing antibodies against VEEV in human sera, suggesting the circulation of this alphavirus in these areas. A higher seropositivity for VEEV was evident in Hone Creek than in the GMA, suggesting a greater distribution of this virus close to Panama. Additionally, the existence of neutralizing antibodies was demonstrated in human samples against alphaviruses other than VEEV and MADV/EEEV. Therefore, it is necessary to conduct further studies to evaluate the circulation of these viruses in other areas of the country, as well as the detection of other alphaviruses that could also be circulating in the country that might be responsible for the “unspecific” neutralizing antibodies obtained in this study. Currently, the diagnosis of arboviruses in any hyperendemic areas, as well as in Costa Rica, represents a public health challenge because the symptoms manifested are usually non-specific (flu-like symptoms); also, they are shared among many arboviruses, even other pathogens; additionally, infected persons might be simply asymptomatic, and infections go unnoticed (5, 8, 10, 30). At the same time, the high endemicity of DENV and the co-circulation of other arboviruses may increase the cross-reactivity in the serological diagnosis, further complicating the diagnosis of alphavirus-infected patients (34, 35). In addition, the lack of specific antiviral treatments or vaccines approved for humans hints at the importance of early diagnosis using reliable laboratory techniques for correct medical handling. Implementing a routine diagnosis for alphaviruses is highly recommended, mainly in those areas with high endemicity to arboviruses.

Furthermore, specific vector control (mosquitoes) for putative alphavirus vectors is recommended. Similarly, repellent and clothing that protects against mosquito bites are advised when entering areas where the enzootic cycle potentially exists. Physical barriers, such as screens on windows or mosquito nets, are highly advisable in homes located within or near these areas (9, 30).

Addressing, preventing, and managing arbovirus outbreaks require the involvement of various public health agencies. It emphasizes the need for a comprehensive, collaborative approach, especially, but not exclusively, at the national level. Persistent challenges in many hyperendemic regions, such as Latin America, include limited resources, the absence of multi-pathogen molecular diagnostic kits for early detection, a shortage of adequately trained clinical and vector control personnel, and low community awareness. These factors remain significant obstacles to an efficient response to arboviral infections in Latin America. These challenges are not exclusive to this geographical region but generally to arbovirus hyperendemic sites such as other tropical and subtropical regions worldwide. In these countries, serological testing for arboviral diseases should be done using PRNT, as it is the only tool where cross-reactive antibodies can be distinguished. We should highlight that the climate crisis might increase the geographical distribution of arboviral diseases due to temperature and precipitation changes, which, in turn, impact viral and vector reproduction and life cycles. Thus, arboviruses and their diagnosis could become a public health issue in other countries in the future.

A broad diagnostic strategy is essential to better understand the clinical and epidemiological landscape during these outbreaks. Such insights can guide the design and implementation of public health interventions and improve epidemiological surveillance efforts, even for alphaviruses, such as MADV/EEEV, which are not considered in traditional molecular surveillance methods because we do not find what we are not looking for.

## Data Availability

The original contributions presented in the study are included in the article/Supplementary material, further inquiries can be directed to the corresponding author.
