# Supplementary material for: Exposure to non-endemic arboviruses (alphaviruses) in Costa Rica assessed from human samples collected in areas with contrasting levels of dengue endemicity
Source: Front Public Health. 2025 Feb 19;13:1537019. doi: 10.3389/fpubh.2025.1537019 (PMC11879952; doi:10.3389/fpubh.2025.1537019)
Supplement: Supplementary file 1 [file Data_Sheet_1.docx]

Supplementary Material

# Supplementary Tables

# Table S1.1. MADV/EEEV and VEEV plaque reduction neutralization test titers for Hone Creek, Talamanca human sera samples.

| Samples | VEEV Titer | EEEV Titer | Positivity |
| --- | --- | --- | --- |
| HC-43 | 1:40 | <1:20 | *Alphavirus* |
| HC-56 | 1:320* | 1:20 | VEEV |
| HC-69 | 1:640* | <1:20 | VEEV |
| HC-76 | 1:80* | <1:20 | VEEV |
| HC-82 | 1:160* | 1/40 | VEEV |
| HC-84 | 1:640* | <1:20 | VEEV |
| HC-85 | 1:160* | <1:20 | VEEV |
| HC-94 | 1:160* | <1:20 | VEEV |
| HC-101 | 1:640* | <1:20 | VEEV |
| HC-109 | 1:20 | <1:20 | *Alphavirus* |
| HC-113 | 1:40 | <1:20 | *Alphavirus* |
| HC-114 | 1:80* | 1:20 | VEEV |
| HC-115 | 1:5120* | <1:20 | VEEV |
| HC-116 | 1:160* | <1:20 | VEEV |
| HC-117 | 1:320* | <1:20 | VEEV |
| HC-121 | 1:20 | <1:20 | *Alphavirus (*>*1:*1280 *for CHIKV)* |
| HC-122 | 1:320* | <1:20 | VEEV |
| HC-127 | 1:5120* | <1:20 | VEEV |
| HC-130 | 1:80* | <1:20 | VEEV |
| HC-135 | 1:320* | <1:20 | VEEV |
| HC-136 | 1:20 | <1:20 | *Alphavirus* |
| HC-137 | 1:320* | <1:20 | VEEV |
| HC-147 | 1:80* | <1:20 | VEEV |
| HC-152 | 1:160* | <1:20 | VEEV |
| HC-153 | 1:160* | <1:20 | VEEV |
| HC-176 | 1:320* | <1:20 | VEEV |
| HC-179 | 1:40 | 1:20 | *Alphavirus* |
| HC-191 | 1:80* | 1:20 | VEEV |
| HC-193 | 1:320* | <1:20 | VEEV |
| HC-195 | 1:80* | <1:20 | VEEV |
| HC-200 | 1:320* | <1:20 | VEEV |
| HC-206 | 1:160* | 1:20 | VEEV |
| HC-207 | 1:320* | 1:20 | VEEV |
| HC-209 | 1:40 | <1:20 | *Alphavirus* |
| HC-210 | 1:80* | <1:20 | VEEV |
| HC-212 | 1:40 | 1:20 | *Alphavirus* |
| HC-218 | 1:20 | <1:20 | *Alphavirus* |
| HC-219 | 1:20 | <1:20 | *Alphavirus* |
| HC-223 | 1:1280* | 1:20 | VEEV |
| HC-225 | 1:160* | <1:20 | VEEV |
| HC-228 | 1:80* | <1:20 | VEEV |
| HC-233 | 1:160* | <1:20 | VEEV |
| HC-236 | 1:320* | <1:20 | VEEV |
| HC-238 | 1:320* | 1:20 | VEEV |
| HC-245 | 1:80* | 1:20 | VEEV |
| HC-246 | 1:40 | <1:20 | *Alphavirus* |
| HC-260 | 1:1280* | <1:20 | VEEV |
| HC-270 | 1:320* | <1:20 | VEEV |
| HC-275 | 1:360* | <1:20 | VEEV |
| HC-285 | 1:80* | <1:20 | VEEV |
| HC-291 | 1:80* | <1:20 | VEEV |
| HC-295 | 1:20 | 1:20 | *Alphavirus* |
| HC-297 | 1:40 | 1/40 | *Alphavirus* |

# * Presence of neutralizing antibodies unequivocally positive against the virus.

# Table S1.2. MADV/EEEV and VEEV plaque reduction neutralization test titers for the Greater Metropolitan Area’s human sera samples.

| Samples | VEEV Titer | EEEV Titer | Positivity |
| --- | --- | --- | --- |
| BS-09 | 1:20 | <1:20 | *Alphavirus* |
| BS-122 | 1:160* | <1:20 | VEEV |
| BS-179 | 1:20 | <1:20 | *Alphavirus* |

# * Presence of neutralizing antibodies unequivocally positive against the virus.

# Supplementary Table S2. Residuals obtained for the Chi-squared tests.

|  | **Alphavirus antibodies** | | **VEEV antibodies** | |
| --- | --- | --- | --- | --- |
| **Sampling site** | Detected | Not detected | Detected | Not detected |
| **Talamanca** | 6.197 | -1.943 | 5.593 | -1.481 |
| **Greater Metropolitan Area** | -5.206 | 1.632 | -4.699 | 1.244 |
